# Low expression of Notch1 may be associated with acute myocardial infarction

**DOI:** 10.3389/fcvm.2024.1367675

**Published:** 2024-05-22

**Authors:** Qing Zhang, Heyu Meng, Xue Wang, Yanqiu Chen, Zhaohan Yan, Jianjun Ruan, Fanbo Meng

**Affiliations:** ^1^ Department of Cardiology, China-Japan Union Hospital of Jilin University, Changchun, Jilin, China; ^2^ Jilin Provincial Engineering Laboratory for Endothelial Function and Genetic Diagnosis of Cardiovascular Disease, Changchun, Jilin, China; ^3^ Jilin Provincial Molecular Biology Research Center for Precision Medicine of Major Cardiovascular Disease, Jilin Provincial Cardiovascular Research Institute, Changchun, Jilin, China; ^4^ Jilin Provincial Precision Medicine Key Laboratory for Cardiovascular Genetic Diagnosis, Changchun, Jilin, China

**Keywords:** acute myocardial infarction, chronic coronary syndrome, Notch1, maker, gene

## Abstract

**Background:**

The transmembrane protein Notch1 is associated with cell growth, development, differentiation, proliferation, apoptosis, adhesion, and the epithelial mesenchymal transition. Proteomics, as a research method, uses a series of sequencing techniques to study the composition, expression levels, and modifications of proteins. Here, the association between Notch1 and acute myocardial infarction (AMI) was investigated using proteomics, to assess the possibility of using Notch1 as a biomarker for the disease.

**Methods:**

Fifty-five eligible patients with AMI and 74 with chronic coronary syndrome (CCS) were enrolled, representing the experimental and control groups, respectively. The mRNA levels were assessed using RT-qPCR and proteins were measured using ELISA, and the results were compared and analyzed.

**Results:**

Notch1 mRNA levels were 0.52 times higher in the peripheral blood mononuclear cells of the AMI group relative to the CCS group (*p* < 0.05) while Notch1 protein levels were 0.63 times higher in peripheral blood plasma in AMI patients (*p* < 0.05). Notch1 levels were not associated with older age, hypertension, smoking, high abdominal-blood glucose, high total cholesterol, and high LDL in AMI. Logistic regression indicated associations between AMI and reduced Notch1 expression, hypertension, smoking, and high fasting glucose.

**Conclusions:**

Notch1 expression was reduced in the peripheral blood of patients with AMI relative to those with CCS. The low expression of Notch1 was found to be an independent risk factor for AMI and may thus be an indicator of the disease.

## 1 Introduction

Cardiovascular disease is a major killer in China, leading to the deaths of approximately 40% of the population (1). This is similar to the situation in the rest of the world (2). Therefore, epidemiological research into its causes and prevention are particularly important.

Coronary artery disease (CAD) represents the most prevalent form of cardiovascular disease. It is caused by luminal stenosis due to various reasons, reducing the blood flow to the myocardium. CAD includes both acute (e.g., unstable angina pectoris) and chronic (e.g., stable angina pectoris and ischemic cardiomyopathy) syndromes.

Acute myocardial infarction (AMI) is a rapidly developing, irreversible and life-threatening acute disease, potentially leading to malignant arrhythmia, cardiogenic shock, cardiac rupture and other serious complications, and is a cause of sudden death (3, 4). Both CCS and AMI are forms of CAD, with CCS having twice the incidence of AMI. There is a close pathophysiological relationship between CCS and AMI. Several common diseases (such as hypertension, diabetes) or risk factors (smoking, alcohol abuse) can cause atherosclerosis of the coronary arteries, resulting in luminal stenosis or obstruction, followed by myocardial ischemia and hypoxia, and resulting in a series of symptoms. If CCS is not treated in time, the walls of the cardiac blood vessels will thicken with the accumulation or rupture of arterial plaques, further reducing the blood volume and potentially leading to the complete occlusion of the lumen. This will tend to aggravate the ischemia and hypoxia in the myocardium, resulting in serious and irreversible necrosis of cardiomyocytes, that is, further myocardial infarction. The prevalence of AMI appears to be increasing. Therefore, the ability to accurately predict AMI would be of great significance to reduce mortality and improve prognosis, which is also the focus of our clinical research.

Tests using peripheral blood are useful for the evaluation of disease (5), as they are non-invasive with easy sample collection, and are widely used in diagnosis, detection of disease progression, and evaluation of treatment effect (6–8). For example, peripheral blood expression levels of AdipoR2 are used to assess coronary atherosclerosis (9), as are those Interleukin-32 (10) and cytokine signaling inhibitor 3 (11).

**Figure 1:**
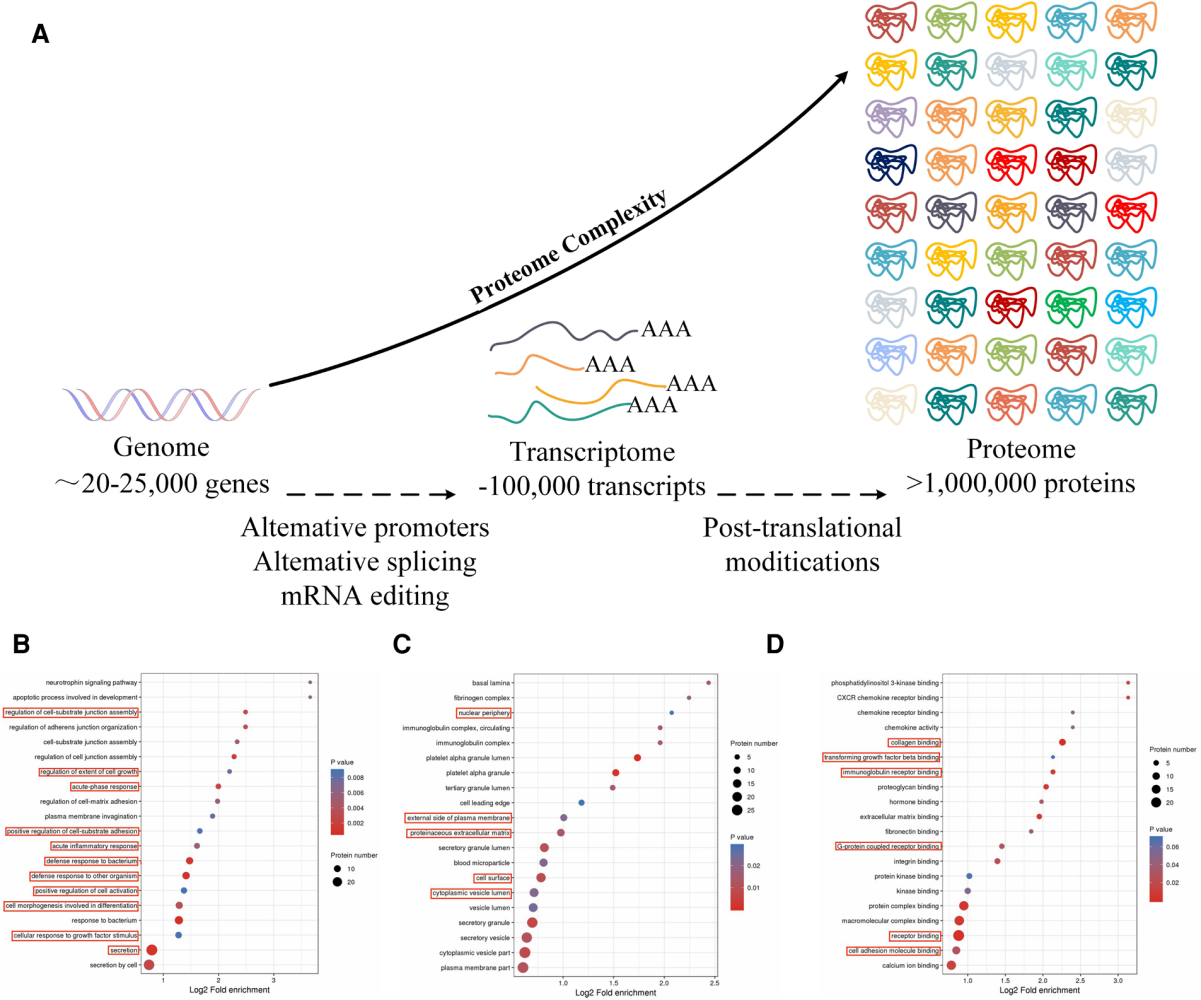
(**A**) Principles of proteomics research: the process from gene to protein. (**B**) The red boxes indicate biochemical processes in which Notch1 is involved. (**C**) In AMI and SCAD, the red boxes indicate cells in which Notch1 is involved in AMI and SCAD. (**D**) The red boxes indicate the molecular functions in which Notch1 is involved in AMI and SCAD.

Here, proteomics was used to identify proteins that were expressed during the early stages of AMI. This showed that Notch1 levels were markedly reduced in AMI patients relative to those with CCS (*p* < 0.05) (Figure 1), suggesting a connection between AMI and reduced Notch1 levels in the peripheral blood. Thus, here, a larger sample size was used to verify the potential association between reduced Notch1 expression in the peripheral blood of AMI patients (12, 13). The accuracy of the proteomics results was also verified by ELISA in the larger sample, and correlation and logistic regression analyses were used to examine the link between Notch1 and AMI and the possibility of using the protein level as an AMI biomarker. These findings may provide new clues for the expression of genes in diseases, and their potential application in diagnosis and treatment.

## 2 Materials and methods

### 2.1 Trial population

This retrospective study enrolled patients who had been treated between September 2017 and April 2019 at the Department of Cardiology, China Japan United Hospital, Jilin University, China. All patients provided informed consent. The experimental group consisted of 55 patients with AMI and control group included 74 patients with CCS. The patients received 12 lead ECG and relevant auxiliary examinations as soon as possible after their first medical contact.

The inclusion criteria for the AMI patients were in accordance with the most recent AMI diagnostic guidelines issued by the European Society of Cardiology in 2017. These were severe stenosis or occlusion of the anterior coronary artery (left/right) or/and other main arteries (anterior descending and circumflex) based on the results of coronary angiography (14, 15). The diagnosis of CCS was determined by a history of previous percutaneous coronary intervention (PCI), coronary artery bypass grafting (CABG), or ≥ stenosis in a minimum of one coronary artery shown on coronary angiography (16). Patients were excluded if they had: 1) myocardial infarction caused by stent thrombosis following PCI or CABG; 2) severe myocarditis and stress heart disease leading to myocardial injury; 3) missing data on important myocardial markers in AMI patients; 4) severe heart failure, pulmonary embolism, aortic dissection (type A or B), and other diseases; 5) myocardial infarction resulting from surgery; 6) secondary myocardial infarction irrespective of cause.

### 2.2 Experiment development

#### 2.2.1 Experimental process

**Figure 2:**
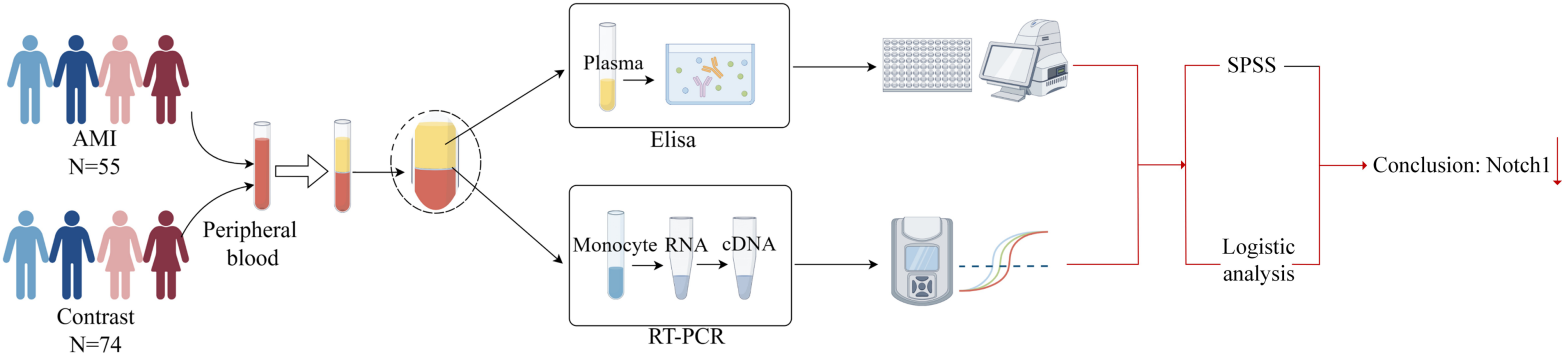
Experimental flowchart.

The experiment used peripheral blood for the study. Patients meeting the above diagnostic criteria were enrolled, with patients with AMI serving as the experimental group and those with CCS as the control group. Notch1 mRNA and protein levels in the AMI group were determined by RT-PCR and ELISA, respectively. Finally, SPSS 25.0 software and logistic regression were applied for analysis (Figure 2).

#### 2.2.2 RT-qPCR

(1) Isolation and processing of peripheral blood mononuclear cells (PBMCs)

Six milliliters of peripheral blood was obtained from participants after an overnight (8 h) fast. The blood was collected into anticoagulant tubes containing EDTA and stored at 4°C before isolation of PBMCs (within 4 h) using Lymphoprep lymphocyte isolation medium (Stemcell Technologies, Vancouver, Canada). Fresh anticoagulant and normal saline were mixed in equal volumes and added to an equal volume of the lymphocyte solution. The mixture was centrifuged at 1,000 g to yield four layers, representing from top to bottom, the plasma, milky white monocyte, clear, and red blood cell layers. The monocyte layer was collected for subsequent studies.

(2) RNA extraction and cDNA acquisition

Total RNA was isolated from PMBCs using a total RNA Extraction Kit (Tiangen Biochemical Technology Co., Ltd., Beijing, China), in strict accordance with the provided directions. RNA concentrations were measured by absorbance using the criteria: ① an A260/A230 value greater than 2; ② an A260/A280 value between 1.7 and 2.1.

The RNA was reverse-transcribed to cDNA using a kit (Tiangen Biochemical Technology Co., Ltd.), following the provided directions, and the cDNA was stored at −80°C before amplification.

(3) RT-qPCR amplification of cDNA

The cDNA samples were diluted 20-fold and amplified using a SYBR Fluorescence Quantification Kit with qPCR synthetic premix (Sangon, Shanghai, China). The reaction conditions were: pre-denaturation at 95°C for 3 min; denaturation at 95°C for 3 s, annealing at 60°C for 30 s, and annealing at 72°C for 20 s, with a total of 40 cycles. The melting and dissociation curves in the range of 60‒95°C were recorded using an ABI-FAST7500 instrument (Applied Biosystems, Waltham, MA, USA). The internal reference was β-actin and relative expression was calculated using the 2^−△△CT^ method. Table 1 shows the primer sequences.

**Table 1:** Primer sequences used for RT-qPCR.

Genes		Genes primer sequence (5’-3’)
Notch1	*F^a^*	GAGGCGTGGCAGACTATGC
	*R^b^*	CTTGTACTCCGTCAGCGTGA
β-action	*F^a^*	TTAGTTGCGTTACACCCTTTC
	*R^b^*	GCTGTCACCTTCACCGTTC

*F^a^*: Upstream primer.

*R^b^*: Downstream primers.

#### 2.2.3 ELISA

(1) Blood collection and processing.

Peripheral blood samples were collected and processed as described above section 2.2.2(1). The top plasma layer obtained after centrifugation was used for ELISA measurements.

(2) An ELISA kit (Shanghai Enzyme-Linked Biotechnology Co., Ltd., Shanghai, China) was used to measure Notch1 protein levels, in accordance with the provided directions. Absorbances at 450 nm were read in a microplate reader and according to the OD value of the standard sample, the linear regression equation of the standard (*R*^2 ^= 0.9953) was calculated. And Notch1 concentrations were assessed in the range of 1.5‒48 ng/ml.

#### 2.2.4 Data processing and analysis

Data were analyzed using SPSS v25.0. After determining the normality of the data distribution, normally distributed data (*p* > 0.1) are expressed as mean ± SD and differences between groups were analyzed by independent samples *t*-tests. Non-normally distributed (*p* ≤ 0.1) data are expressed as median and interquartile range, and differences between groups were analyzed with independent samples rank-sum tests. Count data are presented as frequencies, and differences between groups were analyzed with the chi-square (*χ*^2^) test. Binary logistic regression was used to analyze AMI-related risk factors. Significance levels were defined as two-sided *p* < 0.05.

## 3 Results

### 3.1 Analysis of baseline data

Patient clinical and demographic information was compared in terms of age, hypertension, diabetes mellitus, smoking, drinking, fasting blood glucose, triglycerides, total cholesterol, high-density lipoprotein cholesterol (HDL-C), low-density lipoprotein cholesterol (LDL-C), and white blood cells. Significant differences between the groups were seen in age, blood pressure, smoking, fasting blood glucose, total cholesterol, and LDL-C (*p* < 0.05) (Table 2).

**Table 2:** Baseline information on patients in the AMI and SCAD groups.

Clinical indicators	AMI (*N* = 55)	SCAD (*N* = 74)	*t*/*z*/*x*^2^	*p*
Age	61.000 (54.000–65.250)	51.500 (48.000–54.000)	−6.296	0
Hypertension (%)	34	27	8.122	0.004
Type 2 diabetes (%)	15	12	2.331	0.127
Smoking history (%)	47	48	6.891	0.009
Drinking history (%)	28	30	1.371	0.242
Fasting blood glucose (mmol/L)	7.175 (5.810–9.115)	5.875 (5.175–6.798)	−3.842	0
TG (mmol/L)	1.615 (1.140–2.053)	1.555 (1.170–1.993)	−0.328	0.743
TC (mmol/L)	5.085 (4.263–5.795)	3.600 (3.135–4.505)	−5.172	0
HDL-C (mmol/L)	1.120 (0.965–1.293)	1.040 (0.953–1.230)	−0.681	0.496
LDL-C (mmol/L)	3.462 ± 0.919	2.223 ± 0.704	2.882	0.005
Leukocyte (×10^9^)	9.260 ± 3.463	9.055 ± 3.402	0.153	0.879

### 3.2 Notch1 protein levels

**Figure 3:**
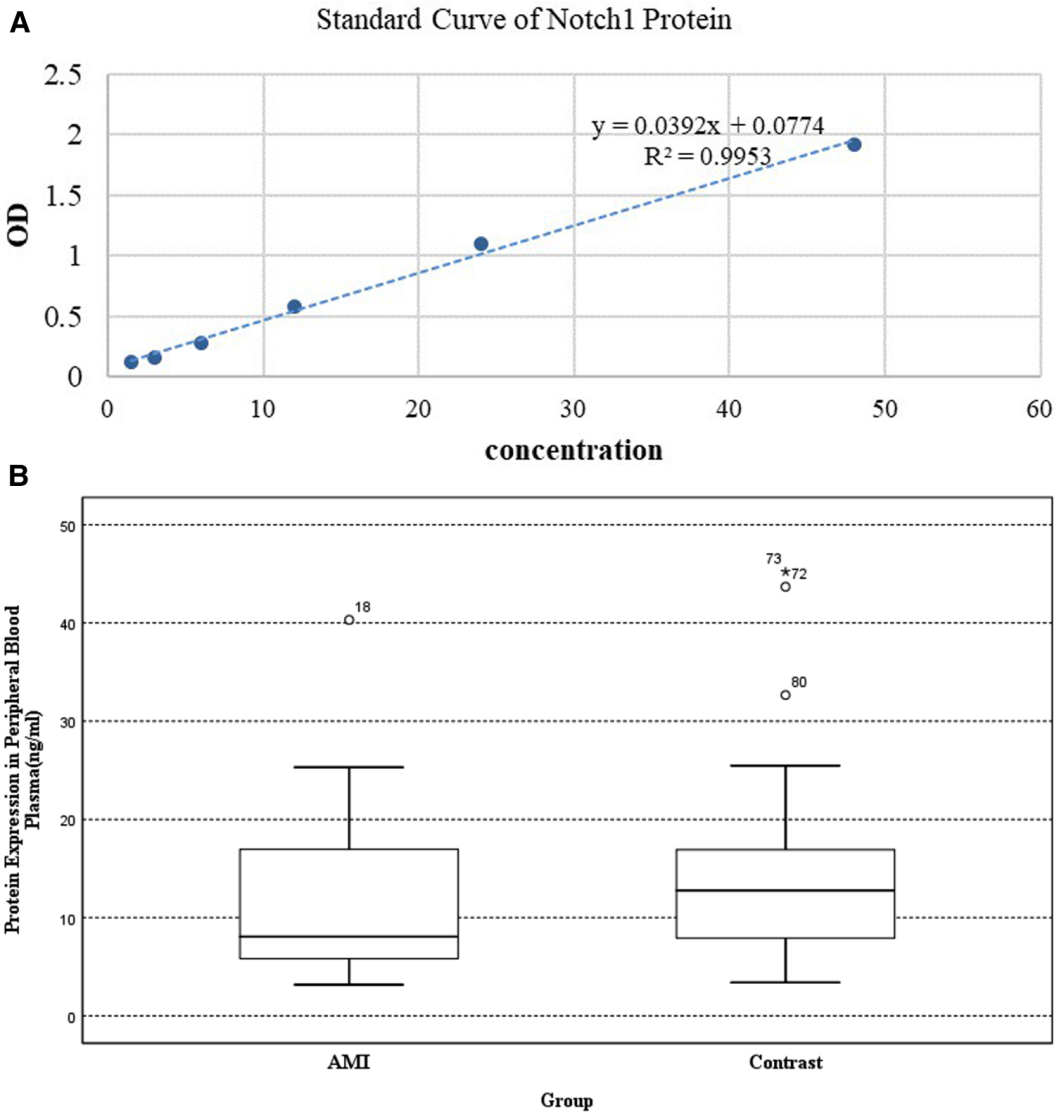
The results of ELISA: (**A**) standard curve of Notch1 protein. (**B**) Expression of Notch1 protein in peripheral plasma.

The Notch1 protein concentrations in AMI patients were found to be 8.058 (5.762–17.190) ng/ml, while those in the CCS group were 12.773 (7.864–17.237) ng/ml. Thus, Notch1 protein levels were 0.63-times higher in experimental group (*p* < 0.05) (Figure 3).

### 3.3 Notch1 mRNA levels

**Figure 4:**
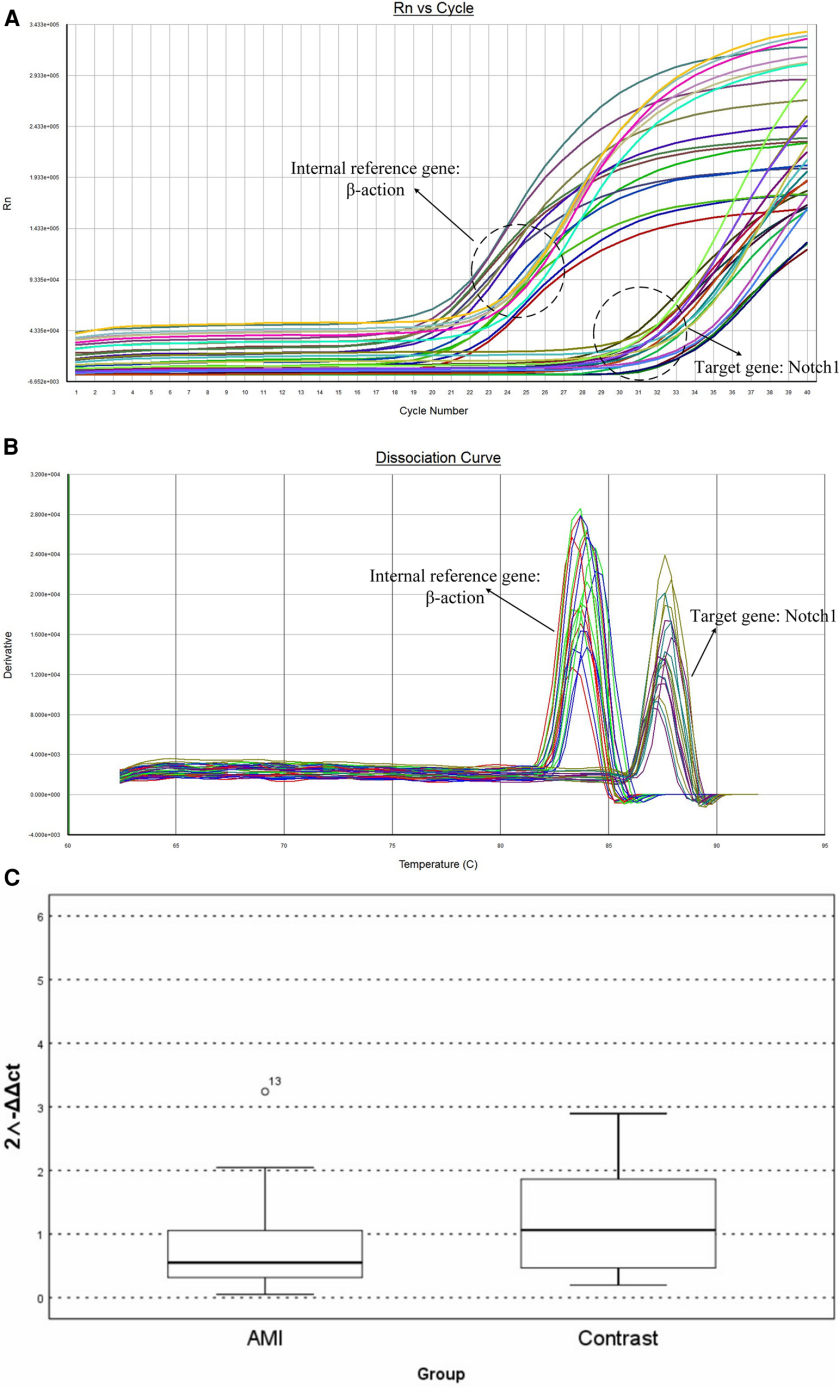
The results of RT-PCR: (**A**) amplification curve of Notch1 gene. (**B**) Dissociation curve of the Notch 1gene. (**C**) Relative mRNA levels in peripheral white blood cells.

*Δ*CT is the average CT value of three consecutive RT-qPCR for each sample. The mRNA expression of the two groups was expressed by 2^−*ΔΔ*CT^. RT-qPCR showed that Notch1 levels were significantly higher (0.52 times, *p* < 0.01) in patients in the AMI group, with the 2^−*ΔΔ*CT^ value of AMI group being 0.552 (0.304–1.172) relative to a value of 1.063 (0.425–1.921) in the CCS group (Figure 4).

### 3.4 Correlation analysis between Notch1 protein expression and sample characteristics

As described above, Notch1 protein levels differed significantly in terms of various demographic and clinical factors, specifically, age, blood pressure, smoking, fasting blood glucose, total cholesterol, and LDL-C between the AMI and CCS groups (*p* < 0.05). Patients in the experimental group were then divided into subgroups according to age (≥65 and <65 years), hypertension and normal blood pressure groups, smoking and non-smoking groups, high fasting blood glucose (≥7 mmol/L) and normal fasting blood glucose (<7 mmol/L) groups, high total cholesterol (≥6.2 mmol/L) and normal cholesterol (<6.2 mmol/L) groups, and high LDL-C (≥4.1 mmol/L) and normal LDL-C (<4.1 mmol/L) groups. Statistical analysis of correlation indicated that Notch1 protein levels were not significantly linked to age, blood pressure, smoking, fasting blood glucose, total cholesterol and LDL (*p* > 0.05) (Table 3).

**Table 3:** Correlation analysis.

Groups	Notch1 expression	*z*	*p*
High age group	9.661 (6.094–17.256)	−0.605	0.545
Young age group	11.561 (6.765–16.918)		
Hypertension Group	10.235 (6.162–16.255)	−1.823	0.068
Normal blood pressure group	13.122 (7.460–17.505)		
Smoking group	10.745 (6.255–16.918)	−1.091	0.275
Non-smoking group	12.552 (7.409–17.926)		
High fasting blood glucose group	11.442 (5.940–16.952)	−1.137	0.256
Normal fasting blood glucose group	10.796 (7.040–17.320)		
High TC group	10.171 (7.237–17.314)	−0.019	0.984
Normal TC group	11.442 (6.527–17.190)		
High LDL-C group	10.235 (6.204–16.714)	−0.245	0.807
Normal LDL-C group	11.145 (6.591–17.193)		

### 3.5 Logistic regression analysis of associations between factors and AMI

The maximum sensitivity and specificity value of 9.457, determined by the correlation between Notch1 levels and AMI, was used as a cutoff (the maximum value corresponding to Youden index, Youden index = sensitivity + specificity - 1). The AMI was divided into subgroups according to Notch1 levels, namely, the high (Notch1 concentration >9.457) and low (concentration ≤ 9.457) groups. Logistic regression analysis showed that low levels of Notch1 protein, hypertension, smoking, and high fasting blood glucose were all associated with AMI. This suggests that reduced levels of Notch1 may be an independent risk factor of AMI, and increase the risk of AMI by 2.604 times (*p* < 0.05). Hypertension, smoking and high fasting blood glucose were also independent risk factors of AMI, increasing the likelihood of AMI by 2.514 times (*p* < 0.05), 3.877 times (*p* < 0.05) and 4.293 times (*p* < 0.01), respectively (Table 4).

**Table 4:** Logistic regression analysis.

	B	Standard variation	Wald	Degree of freedom	*p-*value	OR	95% CI	
Low expression of Notch1	0.957	0.429	4.969	1	0.026	2.604	1.123	6.041
Hypertension	0.922	0.421	4.804	1	0.028	2.514	1.102	5.735
Smoking history	1.355	0.533	6.464	1	0.011	3.877	1.364	11.018
High fasting blood glucose	1.457	0.445	10.702	1	0.001	4.293	1.793	10.276

Because older age, high cholesterol, and high LDL levels had large standard errors in the binary logistic regression analyses, with *P*-values close to 1, these were not included in the logistic regression as it was considered that these were related to the composition ratio of the sample.

## 4 Discussion

It was found that both the mRNA and protein levels of Notch1 were markedly reduced in patients with AMI compared with those with CCS. Specifically, mRNA levels in the AMI group were 0.52 times above those in the CCS group, while protein levels in AMI patients were observed to be 0.52 times than those in CCS patients. Logistic regression analysis indicated that AMI was linked with reduced levels of Notch1, as well with hypertension, smoking, and high levels of fasting glucose. It was also found that this reduced Notch1 expression was an independent risk factor for AMI, and thus may have potential as an indicator for predicting AMI.

Previous proteomics data (17) indicated that protein levels of Notch1 were significantly lower than normal in patients with AMI. While earlier studies have reported the involvement of Notch1 in coronary heart disease, there is little information on the associations of Notch1 levels with either AMI or CCS. Therefore, the present study investigated this potential association in these diseases. To verify the earlier proteomics results, the sample size of the present study was increased, enrolling patients with AMI as the experimental group and those with CCS as the control group. The Notch1 mRNA and protein levels were then evaluated in the peripheral blood of these patients using RT-qPCR and ELISA, respectively. This findings indicated that levels of Notch1 were markedly lower in patients with AMI relative to the CCS patients in the control group (*p* < 0.05), with the relative amounts being 0.52 times and 0.63 times, respectively.

Notch1 was first identified in a mutant gene in Drosophila by T.H. Morgan in 1913 In mammals, Notch genes encode membrane receptor proteins, including four receptors (Notch1‒4), which bind to proteins of the Jagged (Jagged 1 and 2) and Delta (Delta 1, 3, and 4) families (18). The Notch pathway is evolutionarily conserved and is closely involved in various processes, including growth, proliferation, differentiation, cell adhesion, and apoptosis, as well as the epithelial-mesenchymal transition (EMT). Notch1 is a transmembrane receptor protein and functions through binding to specific ligands. It can also be cleaved by proteases, including *γ*-secretase, inducing the release of its intracellular N1ICD structural domain N1ICD, which is then transferred to the nucleus where it functions as a cofactor in regulating the transcription of specific genes (15, 19–21). Although literature has shown that the Notch signaling system exists in myocardial cells and is activated during heart failure, and Notch3 and Notch4 may be the main receptors involved (22), more studies have reported a close relationship between Notch1 and cardiovascular disease. For instance, Wang et al. (23) observed that the Notch1-Jagged1 axis was associated with the EMT in mouse heart tissues, as well as in the early formation of cardiac valves. Mutations in Notch1 have been found to reduce the levels of key factors associated with the EMT, including the transcription factors Heyl, snail, Snai2, Msxl and Msx2, thus causing heart valve disease and aortic calcification (24). Bessa et al. (25) found that Notch1 and RBPJK mutations can lead to impaired formation of trabecular bone, as well as lowering the expression of the Eph receptor-interacting protein B2 (EphrinB2), neuregulin-1 (NRG1), and BMP10. These effects adversely influenced the growth of the myocardium, which can lead to congenital heart disease (CHD) (14). Rentschler et al. (26) found that expression of activated forms of Notch1 during myocardial growth in mice led to ventricular pre-excitation, and that myocardial activation of Notch signaling could influence osmotic pathways associated with ventricular pre-excitation, causing a range of cardiac arrhythmias. Notch1 also provides cardioprotection against myocardial ischemia-reperfusion injury (IRI) by participating in the improvement of mitochondrial quality control (27–29). The Notch1 pathway is also implicated in the physiology of cardiac stem cells (CSCs). CSCs express Notch1 ligands as well as containing activated forms of the N1ICD Notch fragment in the nucleus, and overexpression of N1ICDs significantly expands the number of myocytes. Moreover, a number of experimental studies have shown that blocking the Notch1 pathway in mice with γ-secretase inhibitors increases both the morbidity and mortality associated with dilated cardiomyopathy (30, 31). However, Notch1 is also linked to coronary heart disease, including both AMI and CCS. However, details of the relationship between Notch1 and coronary heart disease require elucidation. Therefore, in this paper, studying two groups of patients with AMI and CCS, respectively, may provide new clues for the identification of genes involved in these diseases, as well as new ideas for early diagnosis and clinical management.

Coronary atherosclerosis (AS) represents the pathophysiological basis of CAD (including AMI and CCS) (32). Although the exact mechanism of AS has not been thoroughly studied, it is known to involve lipogenesis, inflammation, genetic mutation, and receptor deletions. Studies have shown that lipid infiltration, inflammatory responses, endothelial cell injury, oxidative stress, and the cellular activation of smooth muscle in the vasculature are associated with the development of AS. It is also known that certain risk factors, including age, sex, genetics, hyperlipidemia, smoking, and hypertension, amongst others, may exacerbate these processes, resulting in AS aggravation and potential adverse consequences. Therefore, AS is not the result of a single cause, but may be the product of multiple mechanisms and factors.

Vascular endothelial cells are closely involved in the process of AS development. Stimulation or damage to endothelial cells leads to the release of a variety of cytokines that promote the adhesion of inflammatory cells (e.g., monocytes) to the vessel walls, in turn promoting plaque deposition and thus exacerbating the atherosclerotic process. All the Notch1 proteins, together with their Jagged and Delta ligands, are expressed in the vascular system. As described above, Notch1 binds to ligands on neighboring cells, leading to its cleavage and transfer of the N1ICD domain to the nucleus where it modulates transcription (15, 19–21). Many studies have demonstrated the protective function of Notch proteins in vascular diseases. Quillard et al. (33) confirmed the protective actions of Notch2 and Notch4 on endothelial cells in the rat vasculature following heart transplantation. In 2014, Briot et al. (34) showed by a correlative study that in the endothelia of human and mouse vessels, Notch1 is the predominant Notch receptor. Without external activation, inhibition of Notch1 inhibition upregulates the levels of a variety of pro-inflammatory atherosclerotic mediators (IL8, SELE, CXCL1, and TDAG51), resulting in leukocyte binding and potential damage to the vascular endothelium, which leads to or exacerbates the progression of AS.

In recent years, relevant guidelines from several countries have concluded that high levels of LDL are risk factors for cardiovascular events and that lowering these levels can reduce this risk (35–38). When LDL is elevated in the vasculature, it can be easily oxidized to ox-LDL, which can destroy vascular endothelial cells and promote AS. Pro-inflammatory factors associated with AS are derived from, firstly, antigen-presenting cells, for example, macrophages, and secondly, T cells. However, T cells can differentiate into different subtypes under the action of different transcription factors. The main manifestations seen during the development of AS are increases in Th1 cells that promote inflammation and the deficiencies in Th2 that are anti-inflammatory. As normal T cells also express TLR4, ox-LDL can bind to TLR4 on the T-cell surfaces, thereby affecting their biological characteristics. It has been found that inhibition of Jagged1 is inhibited or reduced production of the N1ICD can reduce the levels of IL-4, suggesting that the Jagged1-Notch1 axis promotes Th2 differentiation. This indicates that ox-LDL may upregulate Th1-type cytokines (T-bet) and downregulate Th2-type cytokines (GATA-3) by blocking the expression of Notch1, thereby promoting the inflammatory response and potentially leading to the development of AS (39–42). This would suggest reduced levels of Notch1 in AMI relative to CCS, which is consistent with our experimental results.

Notch1 is downregulated soon after birth and its expression is elevated following cardiac injury, suggesting that this may protect the myocardium (43–45). When IPC and ischemic postconditioning (IPost) processes occur in the myocardium, Notch1 is activated, stabilizing the membrane potential of the mitochondria, decreasing the production of lactate dehydrogenase, reducing IRI-induced reactive oxygen species, limiting the area of myocardial ischemic infarction, and inhibiting apoptosis of cardiomyocytes (46). Notch can upregulate Mfn2 through the NICD/Akt/Mfn cascade effect, leading to mitochondrial fusion, blocking mitochondrial permeability, and reducing myocardial IRI (47); it is also able to downregulate Drp1 expression, thereby inhibiting mitochondrial fission and reducing ischemia in the infarct area (48). Mfn2 can be phosphorylated by Pink1, leading to autophagy via the action of Parkin. Zhou et al. (27) showed that Notch1 can inhibit the Pink1/Parkin interaction by reducing both Pink1 levels and Mfn2 phosphorylation during myocardial IRI, thus reducing mitochondrial autophagy and protecting the damaged myocardium. Gude et al. believe that Notch1 can enhance the activity of Akt in the c-Met and Akt survival signaling pathways, and there is a feedback effect between the two, thereby achieving a protective effect on myocardial cells (49). These results suggest that AMI, which causes myocardial injury, would be associated with increased levels of Notch1, presumably acting as a protective mechanism against the damage. This appears to be the exact opposite of the conclusion we obtained. Possible reasons for this discrepancy may be that: 1. Myocardial IRI is damage that occurs following myocardial infarction when normal perfusion is restored to the myocardium. It is thus different from AMI, and different mechanisms may be involved. 2 The peripheral blood of the subjects enrolled here was collected immediately after the first medical exposure, and the stage of IRI may not have occurred. 3 The pathway of Notch's action in these two diseases is not clear, and requires further investigation.

This retrospective investigation concluded that low expression of Notch1 is associated with AMI, thus suggesting its potential in AMI diagnosis as well as some new ideas and targets for preventing and treating AMI.

However, there are some limitations. The sample size of this experiment is relatively small, and there may be some errors. And no healthy control group was included, which may restrict the application of the findings. Moreover, the underlying mechanism for reduced Notch1 in AMI remains unclear, and we can only rationalize the conclusion based on the original literature. Further investigations into these issues are required.

## 5 Conclusion

Both mRNA and protein levels of Notch1 were observed to be reduced in patients with AMI relative to those with CCS. Low Notch1 expression is an independent risk factor for AMI and may serve as one of the biomarkers for predicting AMI.

## Data Availability

The raw data supporting the conclusions of this article will be made available by the authors, without undue reservation.
